# Supplementary material for: AMBER: Assessment of Metagenome BinnERs
Source: Gigascience. 2018 Jun 8;7(6):giy069. doi: 10.1093/gigascience/giy069 (PMC6022608; doi:10.1093/gigascience/giy069)
Supplement: Supplemental Files [file giy069_supplemental_files.pdf]

Supplementary information for:

AMBER: Assessment of Metagenome BinnERs

Fernando Meyer, Peter Hofmann, Peter Belmann, Ruben Garrido-Oter, Adrian Fritz, Alexander Sczyrba and Alice C. McHardy

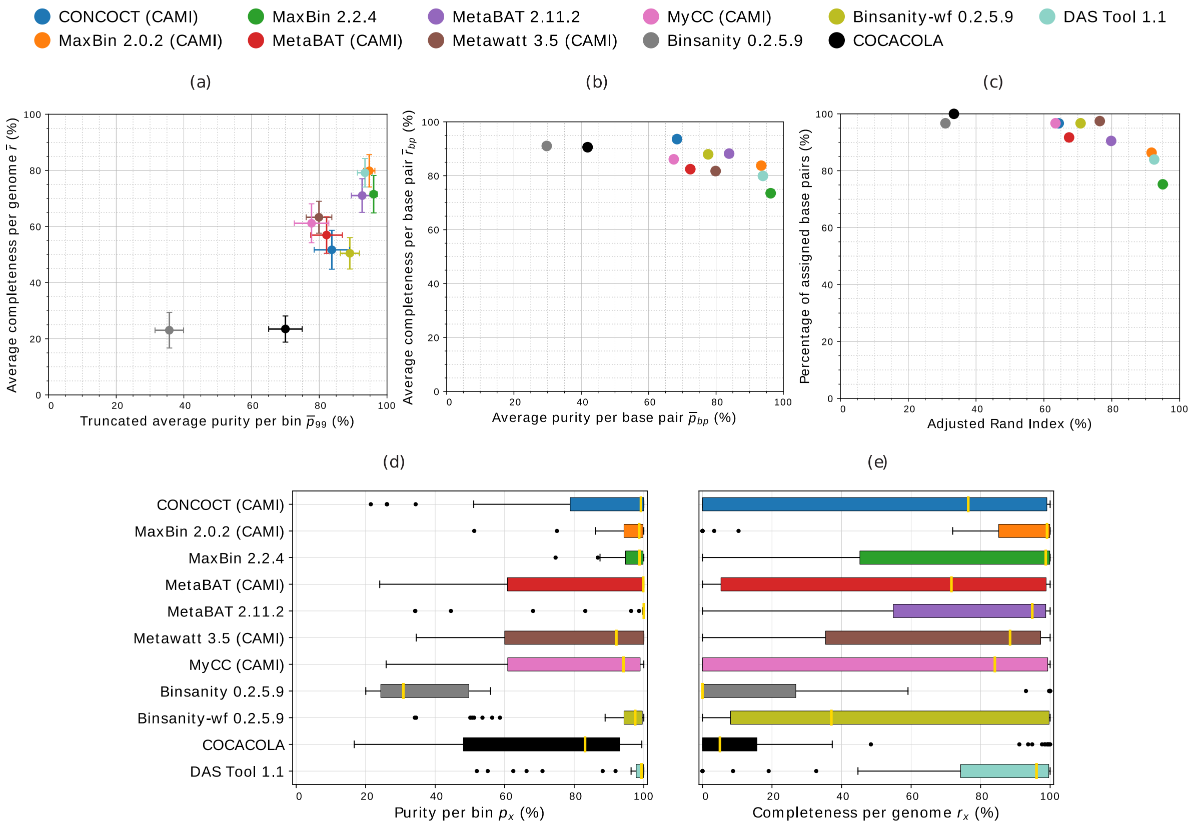

Supplementary Figure 1: Assessment of genomes reconstructed from CAMI's low complexity challenge dataset by different binners. Binner versions participating in CAMI are indicated in the legend in parentheses. (a) Average precision per bin (x-axis), average recall per genome (y-axis), and respective standard errors (bars). As in the CAMI challenge, we report $\overline{\boldsymbol{p}}_{99}$ with 1% of the smallest bins predicted by each program removed. (b) Average precision per base pair (x-axis) and average recall per base pair (y-axis). (c) Adjusted Rand Index per base pair (x-axis) and percentage of assigned base pairs (y-axis). (d-e) Boxplots of precision per bin and recall per genome, respectively.

Supplementary Table 1: Respective number of genomes recovered from CAMI's low complexity data set with less than 10% and 5% contamination and more than 50%, 70%, and 90% completeness.

Genome binner (% contamination)		Predicted bins (% completeness)		
		>50%	>70%	>90%
Gold standard		40	40	40
CONCOCT (CAMI)	<10%	18	17	15
	<5%	16	16	14
MaxBin 2.0.2 (CAMI)	<10%	**28**	**28**	**24**
	<5%	23	**23**	**21**
MaxBin 2.2.4	<10%	24	23	19
	<5%	22	21	18
MetaBAT (CAMI)	<10%	17	16	12
	<5%	17	16	12
MetaBAT 2.11.2	<10%	27	23	20
	<5%	**27**	**23**	20
Metawatt 3.5 (CAMI)	<10%	21	21	16
	<5%	16	16	14
MyCC (CAMI)	<10%	15	15	13
	<5%	13	13	11
Binsanity 0.2.5.9	<10%	0	0	0
	<5%	0	0	0
Binsanity-wf 0.2.5.9	<10%	22	19	18
	<5%	21	18	18
COCACOLA	<10%	2	2	2
	<5%	2	2	2
DAS Tool 1.1	<10%	**30**	**29**	**26**
	<5%	**29**	**28**	**25**

Supplementary Table 2: Total number of bins predicted by each binner on CAMI's high complexity data set and respective number of bins removed to compute the truncated average purity per bin $\bar{p}_{99}$. These are the smallest bins summing up to 1% of the base pairs in all bins. In addition: the respective average purity of the removed bins and the truncated average purity per bin $\bar{\boldsymbol{p}}_{\mathbf{99}}$, as shown in Figure 2 (a) of the main document.

**Genome binner**	**Total number of predicted bins**	**Number of removed bins to compute $\bar{\boldsymbol{p}}_{\mathbf{99}}$**	**Average purity of removed bins**	**Truncated average purity $\bar{\boldsymbol{p}}_{\mathbf{99}}$**
CONCOCT (CAMI)	271	23	0.548	0.693
MaxBin 2.0.2 (CAMI)	342	30	0.596	0.963
MaxBin 2.2.4	331	26	0.597	0.963
MetaBAT (CAMI)	398	58	0.577	0.760
MetaBAT 2.11.2	599	76	0.861	0.914
Metawatt 3.5 (CAMI)	1039	268	0.909	0.903
MyCC (CAMI)	438	65	0.271	0.718
Binsanity 0.2.5.9	122	6	0.970	0.355
Binsanity-wf 0.2.5.9	440	59	0.692	0.776
COCACOLA	513	261	0.308	0.584
DAS Tool 1.1	586	21	0.866	0.931

Steps and commands used to run the assessed binning programs

The following commands were applied to the CAMI high complexity challenge dataset. The commands for the low complexity dataset are very similar and, therefore, omitted.

Download high complexity CAMI dataset from https://data.cami-challenge.org/participate

Preprocessing steps for COCACOLA

Map reads to contigs

/path/to/bowtie2-2.3.2/bowtie2-build -f /path/to/CAMI_high/CAMI_high_GoldStandardAssembly.fasta.gz /path/to/CAMI_high/CAMI_high_GoldStandardAssembly.fasta -p 16
/path/to/bowtie2-2.3.2/bowtie2 -q --fr -x /path/to/CAMI_high/CAMI_high_GoldStandardAssembly.fasta --interleaved /path/to/CAMI_high/RH_S001__insert_270.fq.gz -S /path/to/CAMI_high/RH_S001__insert_270.sam -p 16
/path/to/bowtie2-2.3.2/bowtie2 -q --fr -x /path/to/CAMI_high/CAMI_high_GoldStandardAssembly.fasta --interleaved /path/to/CAMI_high/RH_S002__insert_270.fq.gz -S /path/to/CAMI_high/RH_S002__insert_270.sam -p 16
/path/to/bowtie2-2.3.2/bowtie2 -q --fr -x /path/to/CAMI_high/CAMI_high_GoldStandardAssembly.fasta --interleaved /path/to/CAMI_high/RH_S003__insert_270.fq.gz -S /path/to/CAMI_high/RH_S003__insert_270.sam -p 16
/path/to/bowtie2-2.3.2/bowtie2 -q --fr -x /path/to/CAMI_high/CAMI_high_GoldStandardAssembly.fasta --interleaved /path/to/CAMI_high/RH_S004__insert_270.fq.gz -S /path/to/CAMI_high/RH_S004__insert_270.sam -p 16
/path/to/bowtie2-2.3.2/bowtie2 -q --fr -x /path/to/CAMI_high/CAMI_high_GoldStandardAssembly.fasta --interleaved /path/to/CAMI_high/RH_S005__insert_270.fq.gz -S /path/to/CAMI_high/RH_S005__insert_270.sam -p 16
/path/to/samtools-1.5/bin/samtools view -b -S /path/to/CAMI_high/RH_S001__insert_270.sam -o /path/to/CAMI_high/RH_S001__insert_270.bam
/path/to/samtools-1.5/bin/samtools view -b -S /path/to/CAMI_high/RH_S002__insert_270.sam -o /path/to/CAMI_high/RH_S002__insert_270.bam
/path/to/samtools-1.5/bin/samtools view -b -S /path/to/CAMI_high/RH_S003__insert_270.sam -o /path/to/CAMI_high/RH_S003__insert_270.bam
/path/to/samtools-1.5/bin/samtools view -b -S /path/to/CAMI_high/RH_S004__insert_270.sam -o /path/to/CAMI_high/RH_S004__insert_270.bam
/path/to/samtools-1.5/bin/samtools view -b -S /path/to/CAMI_high/RH_S005__insert_270.sam -o /path/to/CAMI_high/RH_S005__insert_270.bam
/path/to/samtools-1.5/bin/samtools sort -T /path/to/CAMI_high/ -o /path/to/CAMI_high/RH_S001__insert_270-smds.bam /path/to/CAMI_high/RH_S001__insert_270.bam -@ 16
/path/to/samtools-1.5/bin/samtools sort -T /path/to/CAMI_high/ -o /path/to/CAMI_high/RH_S002__insert_270-smds.bam /path/to/CAMI_high/RH_S002__insert_270.bam -@ 16
/path/to/samtools-1.5/bin/samtools sort -T /path/to/CAMI_high/ -o /path/to/CAMI_high/RH_S003__insert_270-smds.bam /path/to/CAMI_high/RH_S003__insert_270.bam -@ 16
/path/to/samtools-1.5/bin/samtools sort -T /path/to/CAMI_high/ -o /path/to/CAMI_high/RH_S004__insert_270-smds.bam /path/to/CAMI_high/RH_S004__insert_270.bam -@ 16
/path/to/samtools-1.5/bin/samtools sort -T /path/to/CAMI_high/ -o /path/to/CAMI_high/RH_S005__insert_270-smds.bam /path/to/CAMI_high/RH_S005__insert_270.bam -@ 16
/path/to/samtools-1.5/bin/samtools index /path/to/CAMI_high/RH_S001__insert_270-smds.bam
/path/to/samtools-1.5/bin/samtools index /path/to/CAMI_high/RH_S002__insert_270-smds.bam
/path/to/samtools-1.5/bin/samtools index /path/to/CAMI_high/RH_S003__insert_270-smds.bam
/path/to/samtools-1.5/bin/samtools index /path/to/CAMI_high/RH_S004__insert_270-smds.bam
/path/to/samtools-1.5/bin/samtools index /path/to/CAMI_high/RH_S005__insert_270-smds.bam

Generate coverage table

python /path/to/CONCOCT/scripts/gen_input_table.py
/path/to/CAMI_high/CAMI_high_GoldStandardAssembly.fasta \
/path/to/CAMI_high/RH_S001__insert_270-smds.bam \
/path/to/CAMI_high/RH_S002__insert_270-smds.bam \
/path/to/CAMI_high/RH_S003__insert_270-smds.bam \
/path/to/CAMI_high/RH_S004__insert_270-smds.bam \
/path/to/CAMI_high/RH_S005__insert_270-smds.bam >
/path/to/CAMI_high/CAMI_high_GoldStandardAssembly.fasta_cov_inputtableR.tsv

Generate composition table

python /path/to/CONCOCT/scripts/fasta_to_features.py
/path/to/CAMI_high/CAMI_high_GoldStandardAssembly.fasta 42038 4
/path/to/CAMI_high/CAMI_high_GoldStandardAssembly.fasta_kmer_4_tmp.csv
42038 is the number of contigs in the FASTA file.

COCACOLA

python cocacola.py --contig_file /path/to/CAMI_high/CAMI_high_GoldStandardAssembly.fasta --abundance_profiles /path/to/CAMI_high/CAMI_high_GoldStandardAssembly.fasta_cov_inputtableR.tsv --composition_profiles /path/to/CAMI_high/CAMI_high_GoldStandardAssembly.fasta_kmer_4_tmp.csv --output /path/to/CAMI_high/cocacola/result_high.csv

MaxBin 2.2.4

./run_MaxBin.pl -thread 16 -contig /path/to/CAMI_high/CAMI_high_GoldStandardAssembly.fasta -out /path/to/CAMI_high/maxbin2/maxbin_cami -reads /path/to/CAMI_high/RH_S001__insert_270.fq -reads2 /path/to/CAMI_high/RH_S002__insert_270.fq -reads3 /path/to/CAMI_high/RH_S003__insert_270.fq -reads4 /path/to/CAMI_high/RH_S004__insert_270.fq -reads5 /path/to/CAMI_high/RH_S005__insert_270.fq

MetaBAT 2.11.2

./runMetaBat.sh /path/to/CAMI_high/CAMI_high_GoldStandardAssembly.fasta /path/to/CAMI_high/RH_S001__insert_270-smds.bam /path/to/CAMI_high/RH_S002__insert_270-smds.bam /path/to/CAMI_high/RH_S003__insert_270-smds.bam /path/to/CAMI_high/RH_S004__insert_270-smds.bam /path/to/CAMI_high/RH_S005__insert_270-smds.bam

BinSanity 0.2.5.9

Binsanity-profile -i /path/to/CAMI_high/CAMI_high_GoldStandardAssembly.fasta -s /path/to/CAMI_high/bam/ --ids /path/to/CAMI_high/CAMI_high_GoldStandardAssembly.fasta.ids -c results.out
Binsanity -f /path/to/CAMI_high -l CAMI_high_GoldStandardAssembly.fasta -c results.out.cov.x100.lognorm
Binsanity-wf -f /path/to/CAMI_high -l CAMI_high_GoldStandardAssembly.fasta -c results.out.cov.x100.lognorm
