# Supplementary material for: AMBER: Assessment of Metagenome BinnERs
Source: Gigascience. 2018 Jun 8;7(6):giy069. doi: 10.1093/gigascience/giy069 (PMC6022608; doi:10.1093/gigascience/giy069)
Supplement: Response_to_Reviewer_Comments_Original_Submission.pdf [file giy069_response_to_reviewer_comments_original_submission.pdf]

Author's Response To Reviewer Comments

Close

Response to Reviewers

As suggested by the Editor, we registered our AMBER software in the SciCrunch.org database and added its Research Resource Identifier SCR_016151 to section "Availability of supporting source code and requirements".

Reviewer #1

Reviewer: While it is relatively easy to access the performance of a given software when using a benchmark dataset with known species composition, the situation is not as straightforward when working with the "unknown". I am afraid that simple users might take these results for granted and only rely on e.g. MetaBAT2 (here proposed as one of the best performing software) in their future studies. While I think that even if it performed better on a simulated dataset (even a very realistic one such as CAMI), how can we be sure that it will work better on any kind of a metagenomics study?

Response: We agree that the reader should not come to the conclusion that MetaBAT 2 is the best program for every dataset and study, and added a discussion along these lines to the Results section. We added the followings sentences to the Results section: “For more extensive information on program performances of multiple data sets, we refer the reader to [1] and future benchmarking challenges organized by CAMI [25].”

Reviewer: Here, the authors have used a recently proposed CAMI dataset that was designed to more realistically mimic common experimental setup, therefore their comparison of the different software might be more correct than previously presented in the separate software articles. Nevertheless, to complement this comparison I would encourage the authors to evaluate in addition the DAS tool by Sieber et al., 2017...

Response: We have addressed this, as suggested. The results for DAS Tool now are included in Figure 2, Supplementary Figure 1, Table 1, and Supplementary Table 2 and discussed in the text. Overall, DAS Tool obtained high quality consensus bins, asserting itself as an option that can be used particularly when is not clear what binner performs best on a specific data set.

Reviewer: ...ICoVeR can visualise and help refine the genome bins resulting from the different software, it does not provide an extensive summary of the binning results in its current version. Therefore, I strongly encourage the authors to further develop in this direction.

Response: We thank the reviewer for these helpful suggestions. We will include a visualization of genome bins similar to ICoVeR's in a future version of AMBER.

Reviewer #2

Major concerns

Reviewer: I have no major concerns with the content of this manuscript. It is well executed and supplies a useful product to the community. I do have some minor comments on some specific language, word choice, and desire a small amount of clarification, discussed below.

Response: Thank you very much for the appreciation of our work.

Minor concerns

Reviewer: Abstract: "ten different binnings" > a better word choice might be "ten different binning tools" or "binning software options"

Response: We replaced "ten different binnings" by "eleven different binning programs" - eleven due to the inclusion of the results for DAS Tool.

Reviewer: Introduction: The use of "populations" in the first sentence probably is not accurate enough. There are many biologist who consider populations in reference to variations in alleles of genes and while that is possible with metagenomic binning techniques, this is probably not what is trying to be addressed. I would remove the word.

Response: We removed the word "populations".

Reviewer: Beyond the title of the manuscript, it is not addressed as to what AMBER stands for - I would include that the first time it is mentioned.

Response: Addressed, as suggested. We added "(Assessment of Metagenome BinnERs)" the first AMBER is mentioned, in the second paragraph of the Introduction.

Reviewer: It would be useful in the introduction to expand on what CAMI used to achieve the goal of assessing metagenomic binning tools previous - this is addressed later, the low and high complexity data sets, but discussing it in the introduction what it is would be helpful.

Response: We added the following explanation to the second paragraph of the Introduction: "Following community requirements and suggestions, the first CAMI challenge provided metagenome data sets of microbial communities with different organismal complexities, for which participants could submit their assembly, taxonomic and genomic binning, and taxonomic profiling results. These were subsequently evaluated, using metrics selected by the community [1]."

Reviewer: Page 8. "If including this unassigned portion into the ARI calculation" > "If this unassigned portion is included into"

Response: Changed, as suggested.

Reviewer: Page 10. Reporting p99 is interesting - is there a specific reason why this cutoff level is used? Is it purely just to illustrate a wide breadth of the binned data? Would be more meaningful for biological interpretation with a higher value? You may want to discuss this decision in the main body of the manuscript. Is there a way of including how many data points are discarded using this metric?

Response: We added the following explanation in the Results section: "As in the evaluation of the first CAMI challenge, we report the truncated average purity, p99, with 1% of the smallest bins predicted by each program removed. These small bins are of little, practical interest for analysis of individual bins and distort the average purity, since their purity is usually much lower than that of larger bins (Supplementary Table 2) and small and large bins contribute equally to this metric."
In the new Supplementary Table 2, we report the number of discarded bins and the average purity of these bins. In addition, we report the total number of bins predicted by each binner and the p99.

Reviewer: Page 11. This is partially addressed at the end of this page: "Notably, some binners, such as CONCOCT, may require more than five samples for optimal performance." I would like to see an expansion on this topic here (or elsewhere) - specifically that default options for software tools may not be the most suitable for all data types. So while the results presented from AMBER here are useful, they should not be taken as an absolute measure of which binner is best, which the authors do not do, but readers likely will. Even some emphasis that different parameters may yield better (or worse) results then presented would be welcome. This case could be made that future researchers should be using AMBER for just this type of analysis!

Response: We agree that parameter settings play a role in the binning performance and that the binners could possibly have performed better. While we do provide AMBER for this kind of analysis, it is only possible when gold standards are available. Hence, we added the following comments to the Results sections: "In general, the binning performance can also be influenced by parameter settings. These could possibly be fine-tuned to yield better results than the ones presented here. We chose to use default parameters or parameters suggested by the developers of the respective binners during the CAMI challenge (Supplementary information), reproducing a realistic scenario where such fine-tuning is difficult due to the lack of gold standard binnings. To thoroughly and fairly benchmark binners, the CAMI challenge encouraged multiple submissions of the same binner with different parameter settings."

Reviewer: Figure 3 suggestions:
In the figure, include the number of target genomes in the dataset.
Provide the number of bins generated for methodology on the x-axis so readers can have that comparison too
Include the units being presented near the scale bar - currently is just says "[millions]" is that recruited reads?

Response: We added the number of genomes and bins on the x and y axes, respectively, counting every third bin and genome due to the limited space. The unit on the scale bar is "millions of base pairs". We changed the figure accordingly.

Close
