# Supplementary material for: AMBER: Assessment of Metagenome BinnERs
Source: Gigascience. 2018 Jun 8;7(6):giy069. doi: 10.1093/gigascience/giy069 (PMC6022608; doi:10.1093/gigascience/giy069)
Supplement: Response_to_Reviewer_Comments_Revision_1.pdf [file giy069_response_to_reviewer_comments_revision_1.pdf]

Author's Response To Reviewer Comments

Close

Response to Reviewers/Editor:

Editor: Your manuscript "AMBER: Assessment of Metagenome BinnERs" (GIGA-D-18-00016R1) has been assessed by Editors and curators, I am pleased to inform you that it is potentially acceptable for publication in GigaScience, once you have carried out some essential formatting revisions for reproducibility purposes. We have taken snapshots of the code in our GigaDB repository so can you check the mockup and let our curators know if you have any changes:
http://gigadb.org/dataset/view/id/100454/token/4aBpyQzsbI0RXCM6
I see you have included a citation to the CAMI benchmarking in GigaDB as well, so we would suggest you also cite this as well in an "Availability of supporting data section" just after the Availability of source code section. This should say something to the following and cite the two references (probably citation 27 for the new DOI)
Availability of supporting data
An archive of the CAMI benchmark data sets [27] and snapshots of the code [28] are available in the GigaScience GigaDB repository.

Response: We added section "Availability of supporting data section", as suggested. However, citation 2 (fixed, as suggested below) already refers to the CAMI benchmark data sets and we decided to reuse it. We did add a new citation (28) for the snapshots of the code, as also suggested below.

Editor: You need to fix the reference to the CAMI dataset to the following (it needs the DOI so people can find the data):
2. Belmann, P; Bremges, A; Dahms, E; Dröge, J; Fiedler, J; Fritz, A; Garrido-Oter, R; Gregor, I; Hofman, P; Janssen, S; Jørgensen, T, S; Koslicki, D; Majda, S; Sczyrba, A; Blood, P, D; Shapiro, N; Gurevich, A; Bai, Y; DeMaere, M, Z; Turaev, D; Chikhi, R; Nagarajan, N; Quince, C; Meyer, F; Balvočiūtė, M; Hansen, L, H; Sørensen, S, J; H. Chia, B, K; Denis, B; Froula, J, L; Wang, Z; Egan, R; Kang, D, D; Cook, J, J; Deltel, C; Beckstette, M; Lemaitre, C; Peterlongo, P; Rizk, G; Lavenier, D; Wu, Y; Singer, S, W; Jain, C; Strous, M; Klingenberg, H; Meinicke, P; Barton, M; Lingner, T; Lin, H; Liao, Y; Z. Silva, G, G; Cuevas, D, A; Edwards, R, A; Saha, S; Piro, V, C; Renard, B, Y; Pop, M; Klenk, H; Göker, M; Kyrpides, N, C; Woyke, T; Vorholt, J, A; Schulze-Lefert, P; Rubin, E, M; Darling, A, E; Rattei, T; McHardy, A, C (2017): Benchmark data sets, software results and reference data for the first CAMI challenge. GigaScience Database. http://dx.doi.org/10.5524/100344

Response: We fixed the reference and added the DOI URL. It is now as follows:
2. Belmann, P., Bremges, A., Dahms, E. et al. Benchmark data sets, software results and reference data for the first CAMI challenge. GigaScience Database (2017). http://dx.doi.org/10.5524/100344.

Editor: You also add the following citation:
27. Meyer, F; Hofmann, P; Belmann, P; Garrido-Oter, R; Fritz, A; Sczyrba, A; McHardy, A, C (2018): Supporting data for "AMBER: Assessment of Metagenome BinnERs" GigaScience Database. http://dx.doi.org/10.5524/100454

Response: Added, as suggested, as reference number 28.

Editor: Please don't cite the URLs or mockups as these are not stable.

Response: We only refer to URLs as described in the Instructions to Authors. We also assigned the following URL, previsously used within the text (page 9, line 17), a reference number:
17. AMBER example HTML visualization of calculated metrics. https://cami-challenge.github.io/AMBER/. Accessed 30 May 2018.

Close
