# Supplementary material for: AMBER: Assessment of Metagenome BinnERs
Source: Gigascience. 2018 Jun 8;7(6):giy069. doi: 10.1093/gigascience/giy069 (PMC6022608; doi:10.1093/gigascience/giy069)
Supplement: Reviewer_1_Report_(Original_Submission) -- Magdalena Calusinska — 1/26/2018 Reviewed [file giy069_reviewer_1_report_(original_submission).pdf]

Reviewer Report

Title: AMBER: Assessment of Metagenome BinnERs

Version: Original Submission **Date:** 1/26/2018

Reviewer name: Magdalena Calusinska

Reviewer Comments to Author:

The manuscript entitled « AMBER : Assessment of Metagenome BinnERs » by Fernando Meyer and collaborators present an evaluation package called AMBER designed to comparatively assess the genome reconstructions for simulated (benchmark) metagenomic datasets. Advancement of massive sequencing technologies promoted, in the last three to four years, development of several binning tools aimed at better re-construction of microbial genomes, including novel species, from environmental metagenomics studies. This has led to recovery of novel genomes of multiple bacteria and archaea, representing in many cases novel phyla; thus increasing our understanding of the so called "microbial dark matter". Even though, most of the state of the art binning tools recently developed combine both the sequence-dependent (e.g. the information about the tetranucleotide composition, number of gene copies of some essential copy genes, GC content etc.) and sequence-independent (e.g. contigs coverage) algorithms, the resulting genome bins might be significantly different. Therefore the development of tools such as AMBER is highly demanded. I could install and run the software on my linux Ubuntu, and the online installation guide and documentation seems quite well done. The authors have compared (extending previous study of CAMI) the accuracy of binning using two types of benchmark datasets (one of high and one of low microbial complexity). As a result, we could clearly see that some software performed better than the others using their specified datasets. While it is relatively easy to access the performance of a given software when using a benchmark dataset with known species composition, the situation is not as straightforward when working with the "unknown". I am afraid that simple users might take these results for granted and only rely on e.g. MetaBAT2 (here proposed as one of the best performing software) in their future studies. While I think that even if it performed better on a simulated dataset (even a very realistic one such as CAMI), how can we be sure that it will work better on any kind of a metagenomics study?My command arises from the fact that in separate articles introducing the different software to the scientific community, the authors proved that their software always performed better than the other tested (often the same set of software were used in the different papers). In addition to the examination of the "unknown" they were always using some benchmark datasets as well. Here, the authors have used a recently proposed CAMI dataset that was designed to more realistically mimic common experimental setup, therefore their comparison of the different software might be more correct than previously presented in the separate software articles. Nevertheless, to complement this comparison I would encourage the authors to evaluate in addition the DAS tool by Sieber et al., 2017, which has been designed to generate a kind of a consensus set of genome bins, based on the separate binning results from different algorithms. It would be of great importance for non-software developers but simple users who often struggle which "binner" should perform better for their specific datasets. If you show that for the CAMI DAS tool can indeed correctly deliver a consensus set of genome bins, it would stimulate other people to not always rely on a single binning software results but to compare the output of the different ones and perhaps merge the results for the optimal bin quality.As a command for a future tool development, I would really like to see a tool like this being able to evaluate my genome bins resulting from the different "binners" for a real environmental metagenomic study. Except for ICoVeR by Broeksema et al., there is no such a tool existing right now. Especially as ICoVeR can visualise and help refine the genome bins resulting from the different software, it does not provide an extensive summary of the binning results in its current version. Therefore, I strongly encourage the authors to further develop in this direction.I strongly recommend this article for publication.

Level of Interest

Please indicate how interesting you found the manuscript: An article of importance in its field

Quality of Written English

Please indicate the quality of language in the manuscript: Acceptable

Declaration of Competing Interests

Please complete a declaration of competing interests, considering the following questions:

- Have you in the past five years received reimbursements, fees, funding, or salary from an organisation that may in any way gain or lose financially from the publication of this manuscript, either now or in the future?
- Do you hold any stocks or shares in an organisation that may in any way gain or lose financially from the publication of this manuscript, either now or in the future?
- Do you hold or are you currently applying for any patents relating to the content of the manuscript?
- Have you received reimbursements, fees, funding, or salary from an organization that holds or has applied for patents relating to the content of the manuscript?
- Do you have any other financial competing interests?
- Do you have any non-financial competing interests in relation to this paper?

If you can answer no to all of the above, write 'I declare that I have no competing interests' below. If your reply is yes to any, please give details below.

I declare that I have no competing interests

I agree to the open peer review policy of the journal. I understand that my name will be included on my report to the authors and, if the manuscript is accepted for publication, my named report including any attachments I upload will be posted on the website along with the authors' responses. I agree for my report to be made available under an Open Access Creative Commons CC-BY license (http://creativecommons.org/licenses/by/4.0/). I understand that any comments which I do not wish to be included in my named report can be included as confidential comments to the editors, which will not be published.

I agree to the open peer review policy of the journal

To further support our reviewers, we have joined with Publons, where you can gain additional credit to further highlight your hard work (see: https://publons.com/journal/530/gigascience). On publication of this

paper, your review will be automatically added to Publons, you can then choose whether or not to claim your Publons credit. I understand this statement.

Yes
