# Supplementary material for: AMBER: Assessment of Metagenome BinnERs
Source: Gigascience. 2018 Jun 8;7(6):giy069. doi: 10.1093/gigascience/giy069 (PMC6022608; doi:10.1093/gigascience/giy069)
Supplement: Reviewer_2_Report_(Original_Submission) -- Benjamin Tully — 2/19/2018 Reviewed [file giy069_reviewer_2_report_(original_submission).pdf]

Reviewer Report

Title: AMBER: Assessment of Metagenome BinnERs

Version: Original Submission **Date:** 2/19/2018

Reviewer name: Benjamin Tully

Reviewer Comments to Author:

SummaryThe authors have developed AMBER, a software package that can be used to generate standardized calculations and visualizations, for assessing the quality of metagenomic binning tools using the CAMI metagenomic data sets, as well as, for implementation on other data sets. The authors implement and define a number measures for these types of assessments as determined by the CAMI community that nicely complement existing methods and then can translate these results to simple visuals. This is definitely a tool that the community needs and is welcomed endeavor.Major concernsI have no major concerns with the content of this manuscript. It is well executed and supplies a useful product to the community. I do have some minor comments on some specific language, word choice, and desire a small amount of clarification, discussed below.Minor concerns(The lack of line numbers may cause some confusion when talking about specific sentences, but we'll survive)Abstract "ten different binnings" > a better word choice might be "ten different binning tools" or "binning software options"IntroductionThe use of "populations" in the first sentence probably is not accurate enough. There are many biologist who consider populations in reference to variations in alleles of genes and while that is possible with metagenomic binning techniques, this is probably not what is trying to be addressed. I would remove the word.Beyond the title of the manuscript, it is not addressed as to what AMBER stands for - I would include that the first time it is mentioned.It would be useful in the introduction to expand on what CAMI used to achieve the goal of assessing metagenomic binning tools previous - this is addressed later, the low and high complexity data sets, but discussing it in the introduction what it is would be helpful.Page 8."If including this unassigned portion into the ARI calculation" > "If this unassigned portion is included into..."Page 10.Reporting p99 is interesting - is there a specific reason why this cutoff level is used? Is it purely just to illustrate a wide breadth of the binned data? Would be more meaningful for biological interpretation with a higher value? You may want to discuss this decision in the main body of the manuscript. Is there a way of including how many data points are discarded using this metric?Page 11.This is partially addressed at the end of this page: "Notably, some binners, such as CONCOCT, may require more than five samples for optimal performance." I would like to see an expansion on this topic here (or elsewhere) - specifically that default options for software tools may not be the most suitable for all data types. So while the results presented from AMBER here are useful, they should not be taken as an absolute measure of which binner is best, which the authors do not do, but readers likely will. Even some emphasis that different parameters may yield better (or worse) results then presented would be welcome. This case could be made that future researchers should be using AMBER for just this type of analysis!Figure 3 suggestions:In the figure, include the number of target genomes in the dataset.Provide the number of bins generated for methodology on the x-axis so readers can have that comparison tooInclude the units being presented near the scale bar - currently is just says "[millions]" is that recruited reads?

Level of Interest

Please indicate how interesting you found the manuscript: An article of importance in its field

Quality of Written English

Please indicate the quality of language in the manuscript: Acceptable

Declaration of Competing Interests

Please complete a declaration of competing interests, considering the following questions:

- Have you in the past five years received reimbursements, fees, funding, or salary from an organisation that may in any way gain or lose financially from the publication of this manuscript, either now or in the future?
- Do you hold any stocks or shares in an organisation that may in any way gain or lose financially from the publication of this manuscript, either now or in the future?
- Do you hold or are you currently applying for any patents relating to the content of the manuscript?
- Have you received reimbursements, fees, funding, or salary from an organization that holds or has applied for patents relating to the content of the manuscript?
- Do you have any other financial competing interests?
- Do you have any non-financial competing interests in relation to this paper?

If you can answer no to all of the above, write 'I declare that I have no competing interests' below. If your reply is yes to any, please give details below.

I declare that I have no competing interests

I agree to the open peer review policy of the journal. I understand that my name will be included on my report to the authors and, if the manuscript is accepted for publication, my named report including any attachments I upload will be posted on the website along with the authors' responses. I agree for my report to be made available under an Open Access Creative Commons CC-BY license (http://creativecommons.org/licenses/by/4.0/). I understand that any comments which I do not wish to be included in my named report can be included as confidential comments to the editors, which will not be published.

I agree to the open peer review policy of the journal

To further support our reviewers, we have joined with Publons, where you can gain additional credit to further highlight your hard work (see: https://publons.com/journal/530/gigascience). On publication of this paper, your review will be automatically added to Publons, you can then choose whether or not to claim your Publons credit. I understand this statement.

Yes
